# Infantile Diarrhea, Fermental Diarrhea; Causes, Symptoms and Treatment*Read before Tri-State Medical Society, Dallas, Texas, 1911. Published in *New Orleans Medical and Surgical Journal*, August, 1911.

**Published:** 1911-09

**Authors:** Joseph W. Gregory

**Affiliations:** Assistant Health Officer, Upshur County, Texas


### · Abstracts and Selections.

### Infantile Diarrhea, Fermental Diarrhea; Causes, Symptoms and Treatment.*

BY DR. JOSEPH W. GREGORY,
Assistant Health Officer, Upshur County, Texas.

In asking indulgence for a short while in presenting a few ideas upon this subject, we do not expect to add to that which has already been written on this well-known variety of diarrheas. We will not attempt to classify this malady under the various heads as described in our text-books, but will offer a few suggestions on the variety commonly seen—towit, the dyspeptic or perverted digestion variety. The prime cause of these diarrheas is attributable to the mal-assimilation of food; hence, we classify under this head.

The subject of infantile diarrheas has always been one of intense interest to the physician whose duty it has been to deal with them. The honest doctor who feels for the welfare of these tender little buds, in directing the best and most practicable methods for their future development from infancy to sturdy man and womanhood, will agree that no affection of childhood gives to the medical man more worry than this class of infantile diarrheas. Statistics bear us out that these bowel disorders in the very young, with their usual complications, carry away more of these little patients than any other class of diseases peculiar to infant life. Infants under the age of thirty months are prone to diarrheal disorders. These troubles foster and flourish during the late spring and summer months. Dentition plays an important role in their cause. The writer, after having a few summers of unpleasant experience with this arch enemy to childhood, began to direct his attention to the causation and subsequent results of treatment, and after some careful investigations into the history, pathology and etiology of these cases, we concluded that the profession had fallen far short of achievements that might have been attained should these conditions have been the subject matter of more sincere research work at the hands of our profession. Our text-book authors have passed the subject by with little mention. Volumes have been written on how to do an appendectomy in the fewest possible moments, or

*Read before Tri-State Medical Society, Dallas, Texas, 1911. Published in *New Orleans Medical and Surgical Journal*, August, 1911.

decapsulate the kidney for nephritis, etc., but few paragraphs we find in our standard works on practice on that monster disease of childhood that snatches from the bosom of a proud parentage more babes than numbers of other diseases combined. It is now known that improper feeding of these children is the cause of this class of troubles. Healthy breast-fed infants are almost immune from this disorder. Authorities agree that only 3 per cent of breast-fed children fall victims to bowel troubles. Just here we desire to digress to condemn in strong terms the popular fad as practiced by our society women who are so unnatural as to refuse their offspring the God-sent privilege of suckling her breast, and use some stratagem to dry up the mammary glands, substituting the dangerous bottle, which furnishes the choicest medium through which to convey diseased germs and toxic material into the intestinal tract of her child. The practitioner who knows his duty and refuses to condemn this practice among the women who expect him to advise them along educational lines, falls far short of doing his sacred duty. His duty is not only to cure the sick, but he should also teach prophylaxis.

As already stated, artificial feeding is the cause of infantile diarrhea. No article of diet appears to be so readily contaminated as cow's milk. It affords an excellent culture medium for almost all forms of bacteria. The nurse prepares the milk for the infant without any regard to cleanliness or hygienic measures, using the rubber nipple and container without sterilizing at each feeding time. The child thus becomes diseased. The doctor often fails here to throw the proper stress on the importance of cleansing everything that the child has to do with. In the healthy breast-fed infants the dejection from the bowels is of a smooth homogeneous nature, of semi-solid, dull lemon color, and not of an unpleasant odor; reaction, acid from the fatty acids contained. The diagnosis of fermental diarrheas then is manifestly easy. The indigestion of the child's food being the prime cause of these disorders, we have classed them as the dyspeptic variety, which classification seems to be justifiable when the frequent ingestion of substances known to be inimical to the normal digestion of the infant's stomach is recalled. This condition, arising from functional derangement of the digestive tract, we will deal first with the milder form, or the acute attack; under this division we usually have the following symptoms: the diarrhea follows after a few days of general malaise, fretfulness and slight pyrexia. It comes on suddenly, with more or less large fluid motions containing fecal matter, traces of blood and undigested material. In some cases, unlike the above, we have symptoms of gastric irritation, abdominal pain which precedes the diarrhea some hours. Examination reveals a moderate amount of fever, 100 to 102 degrees F. In these acute and mild attacks the temperature never runs over the 102 degrees point, except in severe gastric disturbances. The abdomen is usually slightly distended and rounded toward the median line. The stools are frequent, thin and usually sour smell. When the odor is sour we have the condition of acid fermentation. When fetid and very offensive, the albuminous decomposition, the color is pea green, with traces of mucus, and in the ill nourished breast-fed infant the discharge from the bowels is usually also of this peculiar shade. We have often heard the anxious mother, ignorant of the impending danger to her babe, say the child's bowels are acting well, expressing hope in her babe's recovery, saying she had administered oil or teas and the bowels were thus acting.

We will outline our methods of treating the acute cases from a food and therapeutic standpoint. The correction of this condition depends much upon artificial feeding. There is no subject in medicine of which it is more difficult to lay down satisfactory rules to govern all cases than artificial feeding. Eschrich lays down the following rules, recognizing two well known forms of intestinal fermentation, the acid and the alkaline. If there is much decomposition, with foul stools, the albuminous articles should be withheld from the diet, and the carbohydrates given, such as dextrine foods, sugar and milk, which, on account of its sugar, ranks with the carbohydrates. If we have acid fermentation, with sour, but not fetid stools, an albuminous diet should be given, such as broths and egg albumen. Believing the foregoing rules worthy of consideration, we have of recent years adopted Eschrich's principles. If our case is one of acid fermentation we give egg albumen, prepared by a simple method. The whites of two eggs are stirred in warm sterilized water, about a pint, seasoned with salt, and a little brandy is added to flavor. A remarkable improvement of the child fed in this way every one or two hours is soon noticed. Beef juice is also excellent, given alternately with the egg albumen. Boiled water cooled to toleration should be given at frequent intervals, with a small quantity of corn starch. In the albuminous variety we give starch, sugar and sterilized fresh milk and fruit juices. Hygienic measures are not lost sight of. If the pyrexia runs to 102 degrees F. we direct a warm bath, containing a small amount of bicarbonate of soda to aid in cleansing the pores of oily excretions. The cause being one of perverted functional digestion we usually begin therapeutic treatment by hastily getting an evacuation of the bowels, ridding the alimentary tract of any fermenting material by giving minute doses of calomel and ipecac, followed by castor oil. High rectal irrigation with sterilized warm water, containing starch or boric acid, is used. This little operation is best done with a soft rubber No. 10 catheter, which can easily be carried in the emergency bag. Attached to a fountain syringe, using from six to ten ounces, elevating the hips and allowing the enema to remain as long as possible. Two enemas during the twenty-four hours are sufficient. If there is much tenesmus we give paregoric in combination with Resor-Bisnol. It is a safe preparation containing the proper agents, well blended, to take care of the abnormal conditions existing in the alimentary tract. The fermentation and irritation are quickly checked and the patient begins an almost remarkable convalescence. An emulsion is usually prescribed containing Fairchild's Essence of Pepsin. We give it in doses to suit the age and severity of the case every three to four hours. These little patients improve rapidly, the diarrhea ceases and the bowels resume a healthy motion.

We come now to deal with a more severe type of this malady, and will describe it under the caption of a post acute variety. In our modus operandi in dealing with this variety of diarrheas we can not hope for such results as in the acute form. Often we are not called in time to prescribe for these patients until the case has developed into a most serious stage. We will attempt to describe a case of this serious type as seen at the bedside. The pyrexia runs high, often 103 to 105 degrees F., with nervous symptoms manifesting themselves in twitching of the limbs, prostration, wakefulness, head rocking, anxious facies; flabby tissues, depressed fontanelles, sunken, lustreless eyes, with drawn features; the abdomen assumes a flabby feel, the extremities become cold, and within twenty-four hours the algid stage is reached with pallor. The restlessness changes to stupor and an irritable state when disturbed, but rapidly subsides into semi-coma with shallow breathing, collapsed veins, half closed filmy eyes, and terminates by convulsions or coma. In the last described class of cases we are often inclined to diagnose them as cholera infantum, but by careful investigation into the history of these cases leading up to this serious termination, we often obtain a true clinical picture of a once mild attack of intestinal perverted functional disturbance resulting in these unhappy symptoms. Another reason we feel safe in saying our diagnosis is erroneous often when we call this type of the disease cholera infantum, is that, according to the best authors, it is not often met with. So far as ante-mortem clinical evidence goes, cholera infantum may be considered an intense form of acute gastro-enteritis, or a dyspeptic diarrhea plus some unknown intoxication. The best clinicians we have any knowledge of upon these classes of diseases have failed to draw satisfactory and well defined lines between cholera infantum and dyspeptic diarrhea. Cases presenting all the symptoms of so-called cholera infantum from the sudden onset of vomiting to the serious diarrhea seem to yield to treatment. We believe the tendency is to restrict the diagnosis of cholera infantum to fatal cases only, and often the fact is lost sight of by many practitioners of the clinical conditions found early in these cases.

In the routine practice with this serious type of diarrheas we usually follow in part the plan of treatment outlined in dealing with the mild form, but more stress is thrown upon rapid stimulation, supporting the nervous centers and restricting to a thorough antiseptic liquid diet, using Mellin's Food or Horlick's prepared milk. Aconite to control rapid circulation, cold packs for fever, normal salt solution for extreme collapse per rectum, are all useful.

